# Engineered microbial consortia for next-generation feedstocks

**DOI:** 10.1016/j.biotno.2024.01.002

**Published:** 2024-01-17

**Authors:** Gazi Sakir Hossain, Yuanmei Liang, Jee Loon Foo, Matthew Wook Chang

**Affiliations:** a NUS Synthetic Biology for Clinical and Technological Innovation (SynCTI), National University of Singapore, Singapore, Singapore; b Synthetic Biology Translational Research Programme, Yong Loo Lin School of Medicine, National University of Singapore, Singapore, Singapore; c Department of Biochemistry, Yong Loo Lin School of Medicine, National University of Singapore, Singapore, Singapore; d National Centre for Engineering Biology (NCEB), Singapore

**Keywords:** Metabolic engineering, Microbial consortia engineering, Feedstock upcycling, Circular bioeconomy, Synthetic biology

## Abstract

Addressing urgent environmental challenges, this commentary emphasizes the need for green, bio-based solutions in chemical production from renewable feedstocks. It highlights advanced metabolic engineering of microbial strains and the use of microbial consortia as innovative approaches for efficient resource recovery. These strategies aim to enhance the conversion of diverse renewable feedstocks, including agricultural residues, industrial by-products, and greenhouse gases, into value-added chemicals. This article discusses cutting-edge techniques in renewable feedstock upcycling, utilizing both engineered unicellular and multicellular systems. It advocates a paradigm shift in sustainable biomanufacturing, focusing on transforming renewable resources into value-added products. This approach is crucial for developing a circular bioeconomy, aligning with global efforts to mitigate environmental impacts.

## 1 Introduction

In the pursuit of sustainable biomanufacturing, the upcycling of renewable feedstocks into value-added products is increasingly recognized as a critical component of a circular bioeconomy.*^1^* This approach mitigates the environmental impact of waste disposal and contributes to resource conservation and economic growth.*^2^* Among the various technologies for renewable by-product upcycling, microbial-based approaches are particularly noteworthy. These include both single-strain and consortia of multiple strains, each providing distinct and efficacious methodologies. The single-strain-based approach focuses on manipulating the metabolic pathways within a strain to enhance its ability to process both native and non-native feedstocks effectively, transforming them into value-added compounds.*^3^* This field has witnessed substantial advancements, driven by advances in metabolic engineering, and systems and synthetic biology.^4^^,^^5^ On the other hand, the microbial consortium-based approach leverages the synergistic interactions of multiple microbial species to enhance the conversion of feedstocks into value-added products.*^6^* This method addresses some of the intrinsic limitations associated with single-strain-based engineering. It distributes complex metabolic pathways among different strains, optimizing the utilization of diverse renewable feedstock streams.*^7^* These two complementary approaches present compelling opportunities for the transformation of a broad spectrum of renewable materials — encompassing agricultural waste, industrial by-products, and atmospheric greenhouse gases such as CO and CO_2_ into biofuels, pharmaceuticals, and value-added chemical products. In this commentary, we explore the latest advancements, applications, and prospects of metabolic pathway engineering and microbial consortium engineering in the realm of renewable feedstock upcycling, offering insights into their pivotal role in fostering future sustainable biomanufacturing strategies.

## 2 Single-strain metabolic engineering for renewable feedstock upcycling

Metabolic engineering of single strains has emerged as a powerful strategy, allowing precise manipulation of the metabolic pathways of the microorganisms. Unsurprisingly, this approach has also demonstrated an impressive track record in repurposing renewable feedstocks for producing value-added compounds (Table 1). For example, *Escherichia coli*, a heterotrophic organism, was engineered and employed to assimilate CO_2_ feedstock by introducing the Calvin-Benson-Bassham cycle and NAD^+^-coupled formate dehydrogenase. Upon adaptive laboratory evolution, the engineered strain could utilize CO_2_ as the carbon source and formate as the energy source to generate all its biomass. This approach represents a significant advancement in sustainable, value-added chemical production.*^8^* In another example, an engineered *Clostridium autoethanogenum* was used for ethyl acetate production from CO feedstock.*^9^* In addition to industrial emission gas upcycling, recent studies have expanded the realm of lignin repurposing by using not only the white-rot and brown-rot fungi, but also bacteria from the *Proteobacteria*, *Actinobacteria*, *Firmicutes*, and *Bathyarchaeota* phyla.*^10^* This finding highlights the instrumental role of extracellular oxidases like lignin peroxidase and laccase in breaking down lignin into simpler aromatics, ultimately contributing to the tricarboxylic acid cycle, and serving as a testament to nature's intricate and efficient recycling mechanisms.

**Table 1:** Examples of microbial engineering strategies for next-generation feedstock upcycling.

Host microbes	Feedstocks	Strategies of microbial engineering	Products	Ref.
*E. coli*	Carbon dioxide	Introduced the Calvin-Benson-Bassham cycle; expressed an NAD^+^-coupled formate dehydrogenase	Cell biomass	^8^
*C. autoethanogenum*	Carbon monoxide	Heterologous expression of alcohol acetyltransferase	Ethyl acetate	^9^
*S. cerevisiae*	Cellulose and hemicellulose	Expressed and secreted seven lignocellulolytic enzymes; introduced a xylose catabolism pathway	Ethanol	^12^
*C. cellulovorans*	Cellulose	Evaluated 12 different enzyme combinations to determine an efficient n-butanol pathway	n-butanol	^13^
*C. cellulovorans*	Cellulose	Introduced three different aldehyde/alcohol dehydrogenases	Ethanol and n-butanol	^14^
*Y. lipolytica*	Waste cooking oil	Expressed a *cis*-aconitic acid decarboxylase; knocked out genes related to competing pathways	Itaconic acid	^15^
*C. tyrobutyricum*, *V. criceti*, *M. elsdenii*	Cellulose and xylose	Engineered spatial and metabolic niches to switch from a homogeneous to a heterogeneous reactor system	Short-chain fatty acids	^22^
*E. limosum* and *E. coli*	Carbon monoxide	Engineered an *E. coli* strain with biosynthetic pathways and co-cultivating it with *E. limosum*	3-Hydroxypropionic acid and itaconic acid	^23^
*C. glutamicum* and *E. coli*	Chitin	Engineered *E. coli* for lysine auxotrophy and chitin degradation; create synthetic mutualism with *C. glutamicum*	l-lysine	*^26^*

Advances in synthetic biology and genetic tools have also allowed the engineering of cellulolytic strains to use lignocellulose, a key component of plant biomass that is integral to the global carbon cycle and sustainable biofuel production,*^11^* directly as a carbon source. For example, an engineered *Saccharomyces cerevisiae* strain capable of secreting multiple lignocellulolytic enzymes, enabling the direct conversion of lignocellulosic substrates into ethanol without prior enzyme treatment.*^12^* This engineered strain, maintaining robust sugar fermentation performance, promises to significantly reduce enzyme costs in bioethanol production. In another study, the engineering of *Clostridium cellulovorans* to produce ethanol and n-butanol from cellulose at a higher rate demonstrates the potential of using non-conventional strains for renewable feedstock utilization.^13^^,^^14^ Similarly, putting discarded food to good use by employing microbial cell factories has been proven to be feasible and holds great potential. An engineered oleaginous yeast *Yarrowia lipolytica* was used to produce itaconic acid using waste cooking oil by introducing the *cis*-aconitic acid decarboxylase gene from *Aspergillus terreus*, enhancing the acetyl-CoA production pathway and eliminating competing routes.*^15^* Moreover, microbial fermentation technologies are being refined to upcycle unconventional feedstock streams, such as food processing effluents and industrial discharge, overcoming challenges related to impurities, nitrogen content, toxicity, and productivity.*^16^* Furthermore, the burgeoning field of multi-omics technology offers insights into how these independent strains can be genetically engineered to over-produce valuable intermediates to turn renewable feedstock streams into pharmaceuticals, surfactants, chemicals, and polymers.*^17^* As seen in the work on single-strain metabolic engineering for renewable feedstock upcycling, this approach is a highly promising avenue for a sustainable and circular approach to resource recovery and chemical production.

## 3 Microbial consortium engineering for renewable feedstock upcycling

While single-strain metabolic engineering has performed outstandingly to advance renewable feedstock upcycling, the efficiency of feedstock utilization and bioproduction is often still sub-optimal due to the complexity of the biotransformation processes. Therefore, there is increasing interest in recent years to employ microbial consortia to address these limitations by distributing different processes and pathway segments among favoured strains within the community. This approach has proven successful in upcycling various feedstock forms, including agricultural residues and industrial emission gases, showcasing the superiority of microbial consortia in by-product repurposing. The consortium-based multicellular process taps into the collective strength of microbial communities to overcome difficulties in renewable feedstock utilization, such as the degradation and transformation of lignocellulose. Despite the recalcitrant nature of the feedstock, microbial consortia, comprising various strains, efficiently and robustly converted lignocellulose.*^18^* These consortia synergistically deploy diverse enzymes, such as carbohydrate-active enzymes by different strains for cellulose hydrolysis and lignin oxidation.^19^^,^^20^ Interactions like mutualism and commensalism are vital for the consortium-based multicellular process.*^21^* They facilitate the sharing of resources, such as enzymes and nutrients, boosting overall efficiency. For example, an innovative consortium-based approach was used to efficiently transform lignocellulose into short-chain fatty acids.*^22^* This method involves channelling lignocellulosic carbohydrates into lactate as a key intermediate within engineered food chains. The strategy employs a unique spatial niche that facilitates in situ production of cellulolytic enzymes by an aerobic fungus, closely allied with facultative anaerobic lactic acid bacteria and anaerobic bacteria like *Clostridium tyrobutyricum*, *Veillonella criceti*, or *Megasphaera elsdenii*. This integrated approach has demonstrated remarkable efficacy, producing 196 kg of butyric acid per metric ton of beechwood, highlighting a significant advancement in the field of bioconversion and sustainable energy sources (Table 1).

Although the microbial consortia-based approach for renewable feedstock upcycling has mostly been utilizing wild-type strains, the introduction of engineered microbes into a microbial consortium has been demonstrated to contribute to overcoming pressing issues in producing valuable chemicals from renewable resources. Notably, innovative co-cultures consisting of wild-type and engineered strains have been developed to address gas toxicity and by-product issues associated with one-carbon assimilation. By using a combination of *Eubacterium limosum* and engineered *E. coli*, CO has been upcycled for 3-hydroxypropionic acid and itaconic acid production,*^23^* exemplifying the synergy between single-strain metabolic engineering and microbial consortium engineering.

**Fig. 1:**
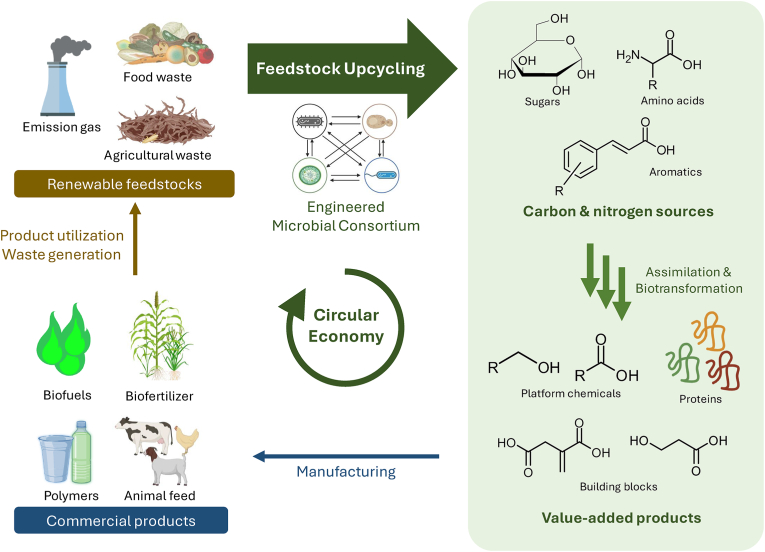
**Next-generation feedstock upcycling using engineered microbial consortia.** The abundance of waste resources provides economical feedstocks for conversion into industrial products. To achieve this, microbial consortia, comprising both wild-type and engineered strains, are employed to facilitate complex processes that produce carbon and nitrogen sources. These are essential for biosynthesizing value-added compounds. Such products can serve as biofuels or be utilized in manufacturing items like polymers, biofertilizers, and animal feeds. By employing feedstock upcycling technologies based on engineered microbial consortia, the subsequent waste generated could again be upcycled, thus promoting a circular bioeconomy.

However, challenges associated with microbial consortium engineering, such as stability and predictability, exist. To overcome these hurdles, synthetic ecology, a fusion of synthetic and computational biology, is instrumental in constructing these microbial consortia.*^24^* Two methods, namely, the "top-down" enrichment from natural settings and "bottom-up" synthetic community assembly, have been employed to cultivate these consortia, which surpass single strains in degradation rates and value-added product production.*^25^* For example, a bottom-up method was used to create a synthetic mutualism between a chitin-metabolizing, lysine auxotrophic *E. coli* strain and a lysine overproducing C*orynebacterium glutamicum* strain.*^26^* This synergistic pairing led to the efficient production of lysine directly from chitin in a single pot bioprocess. Taken together, the integration of engineered microbes into consortia, guided by synthetic ecology principles, holds promise in unlocking new frontiers in the sustainable bioconversion of renewable resources (Fig. 1).

## 4 Future perspective and conclusion

The integration of microbial consortium-based approaches in renewable feedstock upcycling presents a groundbreaking strategy in sustainable bio-manufacturing, aligning with the goals of a circular bioeconomy. The single-strain approach, with its focus on metabolic pathway manipulation within individual strains, has demonstrated significant advancements due to the rapid evolution of synthetic biology and genetic engineering. Conversely, the microbial consortium-based approach utilizes the synergistic potential of multiple microbial species, offering a robust and versatile method for converting diverse renewable feedstock streams into valuable products.

Moving forward, future research needs to focus on optimizing these approaches and maximising their synergy for higher efficiency and scalability. This includes improving the conversion rates of renewable by-product materials and designing systems that can be effectively scaled from the laboratory to industrial levels. In addition, expanding the range of feedstocks that can be repurposed is crucial which includes exploring the potential of these technologies to transform not only agricultural residues, discarded food and industrial by-products but also emerging expended material streams such as used textiles,^27^^,^^28^ rubber*^29^* and plastic.*^30^*

The field of synthetic biology is rapidly evolving, leveraging cutting-edge tools and techniques in genetic engineering, designing genetic biosensor, rewriting metabolic blueprints and incorporating machine learning.^31^, ^32^, ^33^ These advances can lead to the development of more robust and efficient microbial strains capable of processing complex feedstocks. Furthermore, it is essential to assess the economic viability and environmental sustainability of these technologies which involves conducting life cycle analyses and cost-benefit assessments to ensure that these approaches are both environmentally sustainable and economically feasible. More importantly, developing a supportive regulatory framework and fostering public acceptance are key for the successful implementation of renewable feedstock upcycling technologies which can be achieved by addressing safety concerns, ethical considerations, and ensuring transparency in the development and deployment of these technologies.

Finally, the complexity of renewable feedstock upcycling processes demands a collaborative and interdisciplinary approach, involving microbiologists, engineers, environmental scientists, and policymakers. Such collaborations can lead to more innovative solutions and effective implementation strategies. With continued research, innovation, and collaborations among countries where synthetic biology is flourishing,^34^, ^35^, ^36^, ^37^, ^38^, ^39^, ^40^ technologies will undoubtedly be developed to contribute to resource conservation, environmental protection, and the transition towards a more sustainable and circular economy.

## Declaration of competing interest

The authors declare no conflict of interest. Matthew Wook Chang is the Editor-in-Chief for Biotechnology Notes and was not involved in the editorial review or the decision to publish this article.
